# TORC1 INHIBITION AS AN IMMUNOTHERAPY TO DECREASE INFECTIONS IN THE ELDERLY

**DOI:** 10.1093/geroni/igz038.2988

**Published:** 2019-11-08

**Authors:** Joan Mannick

**Affiliations:** resTORbio, Boston, Massachusetts, United States

## Abstract

Inhibition of TORC1 has extended lifespan in multiple preclinical species. Thus drugs inhibiting TORC1 may have therapeutic benefit for aging-related conditions in humans. An aging-related condition that improves with TORC1 inhibition in old mice is immunosenescence (the aging-related decline in immune function). Immunosenescence leads to increased rates of infections including respiratory tract infections (RTIs) in the elderly. In two Phase 2 clinical trials in over 900 elderly subjects, the TORC1 inhibitor RTB101 was observed to improve immune function and decrease the incidence of RTIs. Decreasing the incidence of RTIs is important because RTIs are the fourth leading cause of hospitalization in people age 65 and above. Based on these findings, two Phase 3 studies are underway to determine whether RTB101 given for 16 weeks during winter cold and flu season decreases relative to placebo the percentage of elderly subjects with illnesses associated with RTIs.

